# Shaoyao Gancao Tang (SG-Tang), a formulated Chinese medicine, reduces aggregation and exerts neuroprotection in spinocerebellar ataxia type 17 (SCA17) cell and mouse models

**DOI:** 10.18632/aging.101804

**Published:** 2019-02-13

**Authors:** Chiung-Mei Chen, Wan-Ling Chen, Chen-Ting Hung, Te-Hsien Lin, Ming-Chung Lee, I-Cheng Chen, Chih-Hsin Lin, Chih-Ying Chao, Yih-Ru Wu, Kuo-Hsuan Chang, Hsiu Mei Hsieh-Li, Guey-Jen Lee-Chen

**Affiliations:** 1 Department of Neurology, Chang-Gung Memorial Hospital, Chang-Gung University College of Medicine, Taoyuan 33305, Taiwan; 2 Department of Life Science, National Taiwan Normal University, Taipei 11677, Taiwan; 3 Brion Research Institute, New Taipei City 23143, Taiwan

**Keywords:** spinocerebellar ataxia 17/TBP, Shaoyao Gancao Tang, NFYA, PGC-1α, NRF2, oxidative stress, polyQ aggregates

## Abstract

Spinocerebellar ataxia (SCA) type 17 is an autosomal dominant ataxia caused by expanded polyglutamine (polyQ) tract in the TATA-box binding protein (TBP). Substantial studies have shown involvement of compromised mitochondria biogenesis regulator peroxisome proliferator-activated receptor gamma-coactivator 1 alpha (PGC-1α), nuclear factor erythroid 2-related factor 2 (NRF2), nuclear factor-Y subunit A (NFYA), and their downstream target genes in the pathogenesis of polyQ-expansion diseases. The extracts of *Paeonia lactiflora* (*P. lactiflora*) and *Glycyrrhiza uralensis* (*G. uralensis*) have long been used as a Chinese herbal medicine (CHM). Shaoyao Gancao Tang (SG-Tang) is a formulated CHM made of *P. lactiflora* and *G. uralensis* at a 1:1 ratio. In the present study, we demonstrated the aggregate-inhibitory and anti-oxidative effect of SG-Tang in 293 TBP/Q_79_ cells. We then showed that SG-Tang reduced the aggregates and ameliorated the neurite outgrowth deficits in TBP/Q_79_ SH-SY5Y cells. SG-Tang upregulated expression levels of NFYA, PGC-1α, NRF2, and their downstream target genes in TBP/Q_79_ SH-SY5Y cells. Knock down of NFYA, PGC-1α, and NRF2 attenuated the neurite outgrowth promoting effect of SG-Tang on TBP/Q_79_ SH-SY5Y cells. Furthermore, SG-Tang inhibited aggregation and rescued motor-deficits in SCA17 mouse model. The study results suggest the potential of SG-Tang in treating SCA17 and probable other polyQ diseases.

## Introduction

A group of inherited neurodegenerative diseases including Huntington’s disease (HD), spinobulbar muscular atrophy (SBMA), hereditary spinocerebellar ataxia (SCA) types 1, 2, 3, 6, 7 and 17, and dentatorubral-pallidoluysian atrophy (DRPLA) are caused by an expansion of unstable trinucleotide (CAG) repeats encoding expanded polyglutamine (polyQ) tracts [1]. Among them, SCAs are characterized by cerebellar dysfunction alone or in combination with other neurological abnormalities. SCA17 is an autosomal dominant ataxia caused by an allele containing expanded repeats longer than 43 in the TATA-box binding protein (*TBP*) gene, a transcription initiation factor [2]. In addition to progressive ataxia, the features of this rare disease also include seizure, cognitive dysfunctions, psychiatric symptoms, and pyramidal and extrapyramidal signs such as spasticity, dystonia, chorea, and parkinsonism.

Identification of disease-causative genes has led to the development of model systems for exploration of disease mechanisms and discovery of drug therapy. A prominent pathological feature of the polyQ-mediated diseases is the intranuclear and cytoplasmic accumulation of aggregated polyQ proteins in neurons. Substantial evidence has shown that polyQ-mediated diseases are the result of a toxic gain of function that occurs at the protein level. The presence of expanded polyQ proteins leads to transcriptional dysregulation, mitochondrial damage, oxidative stress, defect in axonal transport, chaperone-proteasome impairment, autophagolysosome dysfunction, and unfolded protein response (UPR) in endoplasmic reticulum (ER) [3,4]. Among them, transcriptional dysregulation is one of the main pathogenic mechanisms of SCA17 [5]. We and Huang et al. have shown that TBP-containing expanded polyQ interacted aberrantly with nuclear factor-Y (NFY) subunit A (NFYA), which would result in reduced heat shock 70 kDa protein 5 (HSPA5) expression [6,7], a major ER chaperone and master regulator of UPR [8]. We have also previously shown downregulation of HSPA5 in lymphoblastoid cells of SCA17 patients, suggesting that decreased ER chaperones may contribute the cell dysfunction of SCA17 [9]. Moreover, elimination of HSPA5 in Purkinje cells leads to accelerated cerebellar degeneration in a mouse model, suggesting an important role of HSPA5 in maintaining neuronal survival [10]. Thus NFY-HSPA5 may serve as a potential target for development of therapeutics for SCA17.

A polyQ mutation can induce reactive oxygen species (ROS) that directly contribute to cell death *in vitro* [11,12] and *in vivo* [13,14]. Antioxidants have been shown to attenuate aggregation and cell death in SCA1, SCA3, and HD models [15–18]. The nuclear factor erythroid 2-related factor 2 (NRF2) and the antioxidant response elements (AREs) signaling pathway is regarded as the major response in the cell to protect against oxidative stress [19]. NRF2 binds to AREs and recruits the general transcriptional machinery for ARE-dependent gene expression when cells respond to oxidative stress. The target genes upregulated by NRF2 including heme oxygenase (decycling) 1 (HMOX1), NAD(P)H dehydrogenase, quinone 1 (NQO1), glutamate-cysteine ligase catalytic subunit (GCLC), and glutathione S-transferase pi 1 (GSTP1) are belonging to the endogenous phase II antioxidative enzymes. Mutant huntingtin and ataxin 3 impaired NRF2 activation and decreased the ARE binding activity, which contributed to mitochondrial dysfunction and enhanced susceptibility to oxidative stress in HD and SCA3 cell models [18,20].

Peroxisome proliferator-activated receptor gamma, coactivator 1 alpha (PGC-1α) is a known regulator of mitochondrial biogenesis and antioxidative response genes including superoxide dismutase 2, mitochondrial (SOD2) and cytochrome c, somatic (CYCS). PGC-1α null mice developed spongiform neurodegeneration in selective brain areas, which suggests the direct role of PGC-1α in neuronal survival [21]. PGC-1α was recently found also to upregulate the NRF2 transcription [22]. Transcriptional repression of PGC-1α by mutant huntingtin resulting in mitochondrial abnormality and neurodegeneration has also been shown in a HD mouse model, suggesting that agents enhancing the transcriptional activity of PGC-1α may be potential therapeutics for HD [23,24]. Indeed, previously we have shown that *Glycyrrhiza inflata* herb extract and its constituents, licochalcone A and ammonium glycyrrhizinate, activated PGC-1α activity and NRF2-ARE signaling to increase mitochondrial biogenesis, decrease oxidative stress, and reduce aggregate formation in SCA3 cellular models [18]. Therefore, we propose that PGC-1α and NRF2 pathways may be also compromised in SCA17 and compounds that enhance PGC-1α and/or NRF2 expression may have potential to treat SCA17.

Shaoyao and Gancao are Chinese herbal medicines (CHMs) prepared from herbs *Paeonia lactiflora* (*P. lactiflora*) and *Glycyrrhiza uralensis* (*G. uralensis*), respectively and have been used to treat oxidative stress, inflammatory, or neurodegenerative disorders [25–29]. Shaoyao Gancao Tang (SG-Tang) is a formulated CHM made of *P. lactiflora* and *G. uralensis* at a 1:1 ratio. SG-Tang inhibits the production of inflammatory cytokines in serum and brain tissue after cerebral ischemia-reperfusion in rats [30]. We have also shown the antioxidative and aggregation-inhibitory effects of SG-Tang in a tauopathy cell model [31]. We therefore examined the effects of SG-Tang on human Tet-On cells with inducible SCA17 TBP/Q_79_-GFP expression, which we have established previously [32]. We also explored if SG-Tang exerts its effect via targeting the PGC-1α/SOD2/CYCS, NRF2/GCLC/ NQO1, and NFYA/HSPA5 pathways. Furthermore, neuroprotective effect of SG-Tang on a previously established SCA17 TBP/Q_109_ transgenic mouse model [33] was investigated.

## RESULTS

### SG-Tang reduced TBP/Q_79_ aggregation and oxidative stress in SCA17 293 cell model

**Figure 1:**
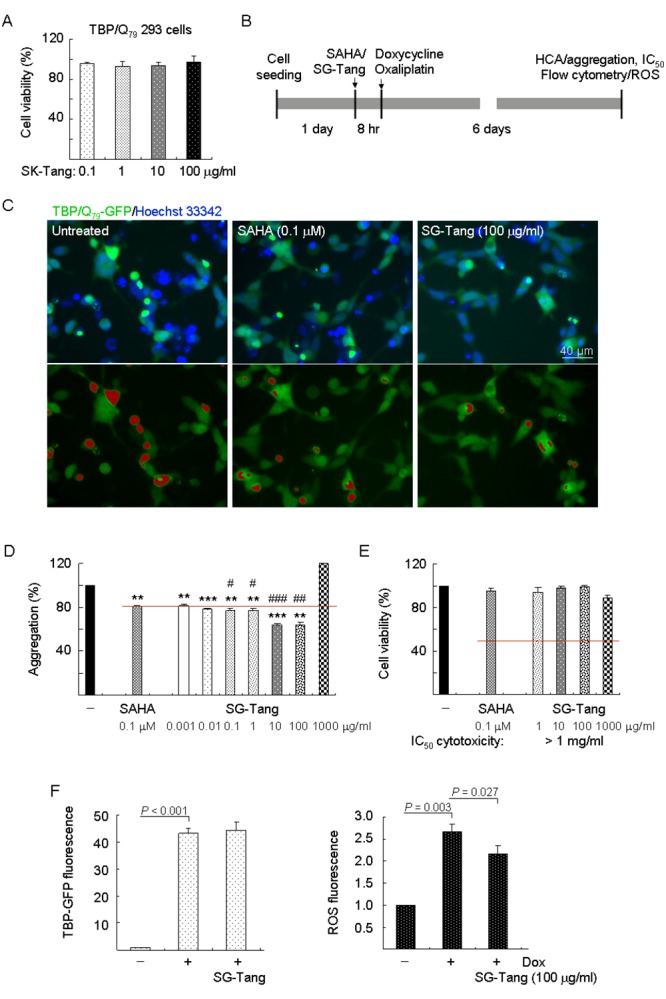
**Aggregation, cytotoxicity, and ROS analyses on TBP/Q_79_-GFP-expressing 293 cells.** (**A**) Cytotoxicity of SG-Tang (0.1−100 μg/ml) in uninduced cells using the MTT assay (*n* = 3). To normalize, the relative untreated cell viability was set as 100%. (**B**) Experimental flow chart. TBP/Q_79_-GFP 293 cells were plated on dishes, grown for 24 h, and treated with SAHA (0.1 μM) or SG-Tang (0.001-1000 µg/ml) for 8 h. Then doxycycline (Dox, 10 µg/ml) and oxaliplatin (5 µM) were added to the medium for 6 days, followed by aggregation, IC_50_ cytotoxicity (by HCA), and ROS (by flow cytometry) measurements. (**C**) Representative fluorescence microscopy images of TBP/Q_79_-GFP cells untreated or treated with SAHA (0.1 μM) and SG-Tang (100 µg/ml) for 6 days, with nuclei counterstained in blue (top row) or aggregates marked in red (bottom row). (**D**) Aggregation analysis (*n* = 3) of TBP/Q_79_-GFP-expressing cells untreated or treated with SAHA (0.1 μM) or SG-Tang (0.001−1000 μg/ml). To normalize, the relative aggregation level in untreated cells was set as 100%. The red line represents 81% aggregation for 0.1 μM SAHA treatment. *P* values: comparisons between SAHA/SG-Tang treated and untreated (**: *P* < 0.01 and ***: *P* < 0.001), or between SG-Tang treated and SAHA treated (^#^: *P* < 0.05, ^##^: *P* < 0.01 and ^###^: *P* < 0.001). (**E**) IC_50_ cytotoxicity of SG-Tang (1−1000 μg/ml) in induced TBP/Q_79_-GFP 293 cells by the percentage of survived cells (*n* = 3). To normalize, the relative survived cell number in untreated cells was set as 100%. (**F**) The induced GFP and ROS levels were measured by flow cytometry (*n* = 3). *P* values: comparisons between induced and uninduced cells, or between SG-Tang (100 µg/ml) treated and untreated cells.

Firstly, TBP/Q_79_-GFP 293 cells were used to evaluate cytotoxicity of SG-Tang. MTT viability test revealed no significant toxic effect on cell survival during 24-h incubation of SG-Tang (97%–93% for 0.1–100 μg/ml treatment) (Figure 1A). To further test the polyQ aggregation-inhibitory and ROS-reducing effects of the SG-Tang, the TBP/Q_79_-GFP cells were treated with SG-Tang (0.001–1000 μg/ml) or histone deacetylase inhibitor SAHA (0.1 μM, as a positive control) [34] for 8 h and induced TBP/Q_79_-GFP expression (by doxycycline) under cell division inhibition (by oxaliplatin) for 6 days (Figure 1B). Representative microscopy images of TBP/Q_79_-GFP aggregation in untreated or SAHA (0.1 μM) or SG-Tang (100 µg/ml) treated cells were shown in Figure 1C. SAHA at 0.1 µM significantly reduced the TBP/Q_79_-GFP aggregation to 81% (*P* = 0.001) compared with untreated cells (100%) (Figure 1D). Treatment of SG-Tang at 0.001–100 µg/ml also significantly reduced the TBP/Q_79_-GFP aggregation (81%–64%, *P* = 0.003 to <0.001). In addition, aggregation-inhibitory effect of SG-Tang at 0.1-100 µg/ml was significantly better than that of SAHA at 0.1 µM (77%–81%, *P* = 0.041 to <0.001). In 293 cells expressing TBP/Q_79_-GFP, SG-Tang had an IC_50_ greater than 1 mg/ml (Figure 1E), indicating its very low cytotoxicity on polyQ-expanded 293 cells.

Abnormal TBP-containing polyQ expansion has been shown to increase cellular ROS level [32]. To evaluate whether SG-Tang reduced oxidative stress in TBP/Q_79_-GFP 293 cells, the cellular ROS production was measured. As shown in Figure 1F, significantly increased ROS production (267%, *P* = 0.003) was observed in cells with induced TBP/Q_79_-GFP expression (+ Dox) for 6 days (43.2-fold expression, *P* < 0.001). With the similar induced green fluorescence (44.4-fold, *P* > 0.05), SG-Tang significantly ameliorated oxidative stress induced by TBP/Q_79_-GFP expression (ROS fluorescence reduced from 267% to 217%, *P* = 0.027).

### SG-Tang reduced TBP/Q_79_ aggregation and promoted neurite outgrowth in SCA17 SH-SY5Y cell model

**Figure 2:**
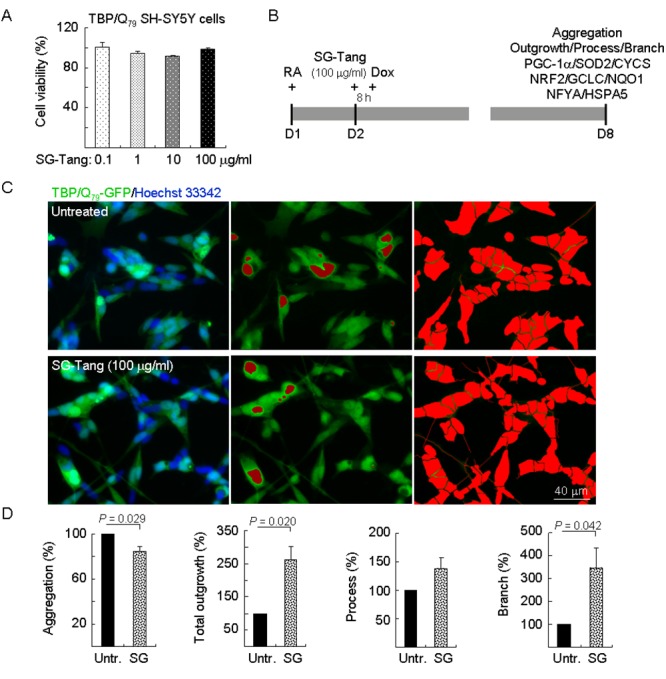
**Neuroprotective effects of SG-Tang in TBP/Q_79_-GFP-expressing SH-SY5Y cells.** (**A**) Cytotoxicity of SG-Tang (0.1−100 μg/ml) in uninduced cells using the MTT assay (*n* = 3). To normalize, the relative untreated cell viability was set as 100%. (**B**) Experimental flowchart. TBP/Q_79_-GFP SH-SY5Y cells were plated on dishes with retinoic acid (RA, 10 µM) added at day 1 to initiate neuronal differentiation. Next day, SG-Tang (100 μg/ml) was added to the cells for 8 h followed by inducing TBP/Q_79_-GFP expression (+ Dox, 5 µg/ml) for 6 days. Aggregation, neurite outgrowth, process, and branch were assessed by HCA. In addition, relative PGC-1α, NRF2, NFYA, and downstream targets were analysed by immunoblotting using specific antibodies. (**C**) Representative microscopic images of differentiated TBP/Q_79_-GFP-expressing SH-SY5Y cells untreated (top row) or treated with SG-Tang (bottom row), with nuclei counterstained in blue (left) or aggregates marked in red (middle). Shown right were images of the neurites and cell bodies outlined by red color for outgrowth quantification. (**D**) Relative aggregation, neuronal outgrowth, process, and branch of TBP/Q_79_-GFP-expressing SH-SY5Y cells with SG-Tang treatment (*n* = 3). To normalize, the relative aggregation, outgrowth, process, or branch level in untreated cells was set as 100%. *P* values: comparisons between treated and untreated cells.

Again, TBP/Q_79_-GFP SH-SY5Y cells were firstly used to evaluate cytotoxicity of SG-Tang. MTT viability test revealed no significant toxic effect on cell survival during 24-h incubation of SG-Tang (101%–91% for 0.1–100 μg/ml treatment) (Figure 2A). To further examine the aggregation-reducing potential of SG-Tang in neuronal cells, TBP/Q_79_-GFP SH-SY5Y cells were differentiated using retinoic acid [35] for one week (Figure 2B). Representative images of TBP/Q_79_-GFP cells untreated or treated with SG-Tang (100 µg/ml) are shown in Figure 2C (Supplementary Figure 1). Treatment with SG-Tang led to 16% reduction of aggregation (*P* = 0.029) in TBP/Q_79_-GFP-expressing neuronal cells (Figure 2D). In addition, significantly increased neurite outgrowth was observed with SG-Tang treatment (total outgrowth: 262%, *P* = 0.020; branch: 346%, *P* = 0.042) (Figure 2D). These results demonstrate the aggregation-inhibitory and outgrowth-promoting effects of SG-Tang in differentiated neurons expressing TBP/Q_79_-GFP.

### Molecular targets of SG-Tang in SCA17 SH-SY5Y cell model

**Figure 3:**
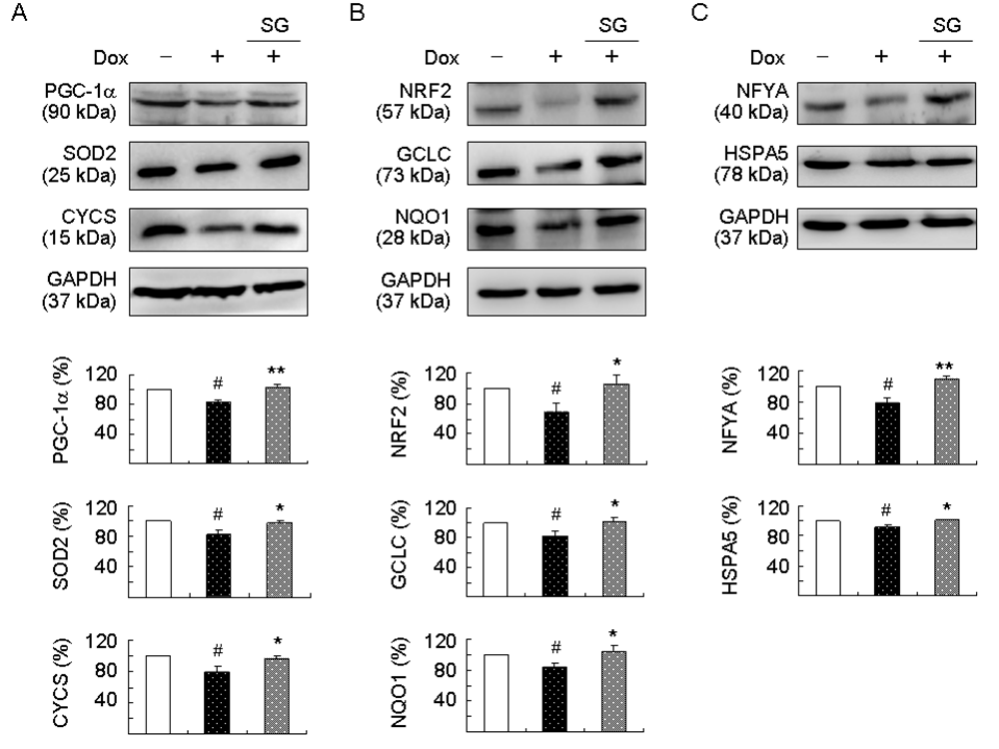
**Enhanced expression of PGC-1α, NRF2, and NFYA pathways following SG-Tang administration on TBP/Q_79_-GFP-expressing SH-SY5Y cells.** On day 2, differentiated SH-SY5Y cells were pretreated with 100 μg/ml SG-Tang for 8 h and TBP/Q_79_-GFP expression was induced for 6 days. Relative (**A**) PGC-1α, SOD2, and CYCS; (**B**) NRF2, GCLC, and NQO1; and (**C**) NFYA and HSPA5 protein levels were analysed by immunoblotting using specific antibodies. Levels of protein were normalized to GAPDH internal control (*n* = 3). Relative protein levels were shown below the representative Western blot images. To normalize, expression level in uninduced (without Dox) cells was set at 100%. *P* values: comparisons between induced and uninduced cells (^#^: *P* < 0.05), or between treated and untreated cells (*: *P* < 0.05 and **: *P* < 0.01).

PGC-1α is a regulator of PPARγ in the brain playing a positive role in ROS suppression [36]. NRF2 is a master regulator of the antioxidant response through regulating expression of phase II antioxidant and detoxification genes [37]. In SCA3 ATXN3/Q_75_ cells, expressions of PGC-1α, NRF2, and their downstream SOD2, CYCS, GCLC, and NQO1 were reduced in response to expanded polyQ stress [18]. In addition, NFYA and HSPA5 expressions were reduced in SCA17 TBP/Q_79_ cells [6]. NFYA is a regulatory subunit of NFY modulating HSPA5 expression in reaction to misfolded proteins in the ER. Thus, we examined the expression of PGC-1α, NRF2, NFYA, and their downstream targets by immunoblotting using specific antibodies. Induced expression of TBP/Q_79_-GFP in differentiated SH-SY5Y cells significantly attenuated the expression of PGC-1α and the downstream SOD2 and CYCS (83%–79% of control, *P* = 0.039–0.010). This attenuation was rescued by the treatment with SG-Tang (increased to 96%–102%, *P* = 0.039–0.008) (Figure 3A). In addition, induction of TBP/Q_79_-GFP significantly reduced NRF2 and the downstream GCLC and NQO1 expression (69%–84% of control, *P* = 0.045–0.036). This reduction was also rescued by the treatment with SG-Tang (increased to 100%–105%, *P* = 0.036–0.022) (Figure 3B). For NFYA and downstream HSPA5, induced expression of TBP/Q_79_-GFP led to 8%–21% reduction (*P* = 0.039–0.029) and the reduction was ameliorated by the treatment with SG-Tang (increased to 110%–101%, *P* = 0.005–0.033) (Figure 3C).

### PGC-1α, NRF2, and NFYA as therapeutic targets in SCA17 SH-SY5Y cell model

**Figure 4:**
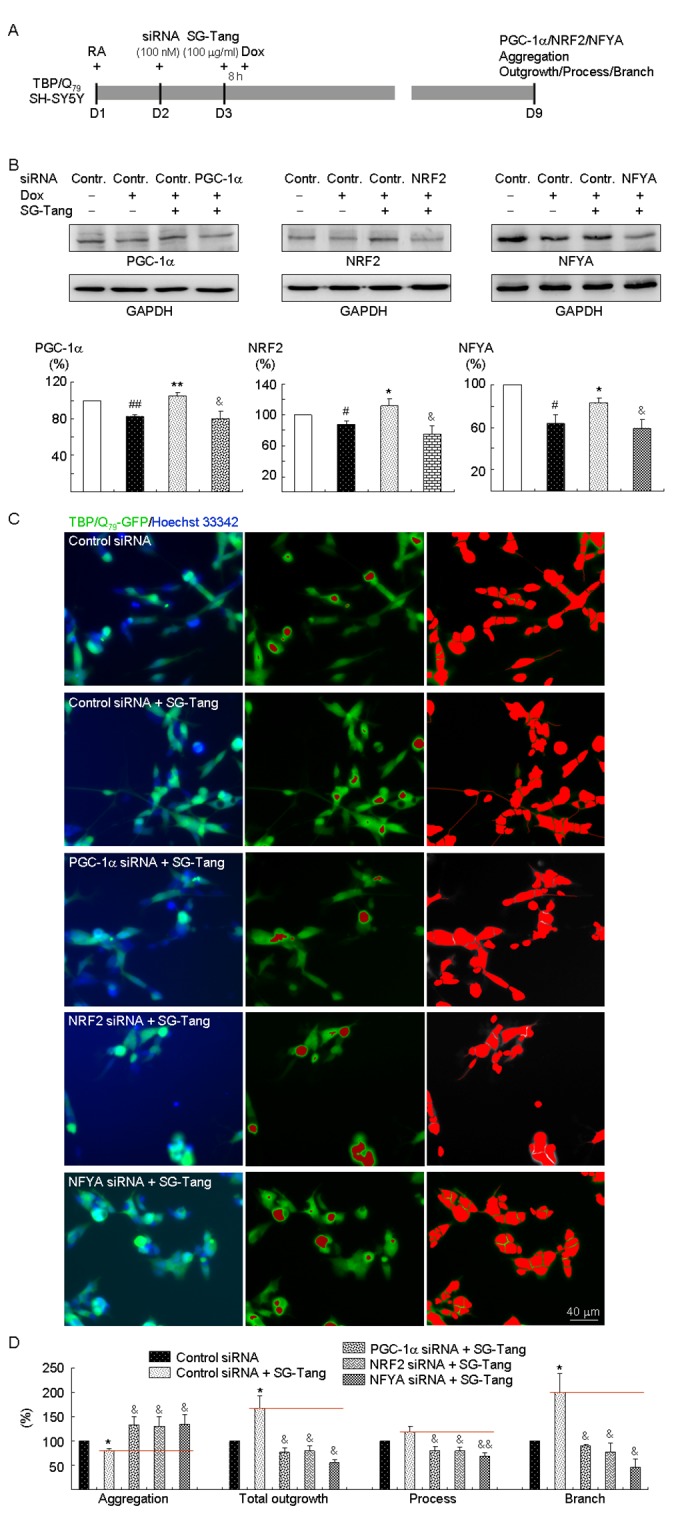
**PGC-1α, NRF2, and NFYA as therapeutic targets in SG-Tang-treated TBP/Q_79_-GFP-expressing SH-SY5Y cells.** (**A**) Experimental flowchart. TBP/Q_79_-GFP SH-SY5Y cells were plated on dishes with retinoic acid (RA, 10 µM) added at day 1. Next day, cells were transfected with siRNA (100 nM; PGC-1α, NRF2, NFYA, or scrambled control). At 24 h post-transfection, SG-Tang (100 µM) was added to the cells for 8 h followed by inducing TBP/Q_79_-GFP expression (+ Dox, 5 µg/ml) for 6 days. Then, the cells were collected for PGC-1α, NRF2, and NFYA protein analysis (by Western blot, GAPDH as a loading control), or stained with Hoechst 33342 for aggregation, neurite outgrowth, process, and branch analyses (by HCA). (**B**) Western blot analysis of PGC-1α, NRF2, and NFYA protein levels in SG-Tang-treated cells transfected with PGC-1α, NRF2, NFYA, or scrambled control siRNA. To normalize, the relative PGC-1α, NRF2, or NFYA level of uninduced cells was set as 100%. *P* values: induced versus uninduced cells (^#^: *P* < 0.05 and ^##^: *P* < 0.01); SG-Tang-treated versus untreated cells (*: *P* < 0.05 and **: *P* < 0.01); or PGC-1α, NRF2, or NFYA siRNA versus scrambled control siRNA-transfected cells (^&^: *P* < 0.05) (*n* = 3). (**C**) Representative microscopic images of TBP/Q_79_-GFP-expressing SH-SY5Y cells transfected with scrambled control siRNA and SG-Tang-treated cells transfected with scrambled control, PGC-1α, NRF2, or NFYA siRNA. Nuclei were counterstained with Hoechst 33342 (blue, left) and aggregates were marked in red (middle). Shown left are images of the neurites and cell bodies outlined by red color for neurite outgrowth, process, and branch quantification. (**D**) Aggregation and neuronal outgrowth, process, and branch assays of SG-Tang-treated TBP/Q_79_-GFP-expressing SH-SY5Y cells transfected with scrambled control, PGC-1α, NRF2, or NFYA siRNA. To normalize, the relative aggregation, outgrowth, process, or branch of scrambled control siRNA-transfected cells without SG-Tang treatment was set as 100%. *P* values: SG-Tang-treated versus untreated cells (*: *P* < 0.05); PGC-1α, NRF2, or NFYA siRNA versus scrambled control siRNA-transfected cells (^&^: *P* < 0.05 and ^&&^: *P* < 0.01) (*n* = 3).

Finally, we explored the effects of PGC-1α, NRF2, and NFYA gene knockdown in aggregation and neurite outgrowth by sequence-specific silencing of the target mRNA molecule. TBP/Q_79_-GFP SH-SY5Y cells were transfected with PGC-1α-, NRF2-, and NFYA-specific, or scrambled control siRNA. The next day, cells were pretreated with SG-Tang for 8 h followed by doxycycline-induced TBP/Q_79_-GFP expression for 6 days (Figure 4A). When the relative PGC-1α, NRF2, or NFYA level of uninduced cells transfected with scrambled control siRNA was set as 100%, induced expression of TBP/Q_79_-GFP reduced the expression of PGC-1α, NRF2, and NFYA (88%–63% versus 100%, *P* = 0.044–0.005) and treatment with SG-Tang significantly increased PGC-1α, NRF2, and NFYA levels (111%–83% versus 88%–63%, *P* = 0.040–0.003). Compared with scrambled control siRNA-transfected cells, PGC-1α, NRF2, and NFYA-specific siRNA significantly attenuated PGC-1α, NRF2, and NFYA levels, respectively, in SG-Tang-treated cells (80%–59% versus 111%–83%, *P* = 0.021–0.013) (Figure 4B). Accordingly, SG-Tang treatment reduced expanded polyQ aggregation (from 100% to 80%, *P* = 0.026) and improved neurite total outgrowth (from 100% to 166%, *P* = 0.032), process (from 100% to 118%, although not significant), and branch (from 100% to 200%, *P* = 0.039) in TBP/Q_79_-GFP-expressing cells transfected with scrambled control siRNA. PGC-1α, NRF2, or NFYA siRNA significantly counteracted the effects of SG-Tang on TBP/Q_79_-GFP-expressing SH-SY5Y cells by increasing expanded polyQ aggregation (131%–135% versus 80%, *P* = 0.036–0.023) and reducing neurite total outgrowth (79%–55% versus 166%, *P* = 0.019–0.014), process (80%–70% versus 118%, *P* = 0.013–0.008), and branch (90%–47% versus 200%, *P* = 0.038–0.011) compared with the scrambled control (Figure 4C and 4D, Supplementary Figure 2). The results showed that the beneficial effects of SG-Tang were attenuated, but not abolished by the knockdown of PGC-1α, NRF2, or NFYA.

### SG-Tang ameliorated behavioral deficits and reduced aggregation in SCA17 TBP/Q_109_ TG mice

**Figure 5:**
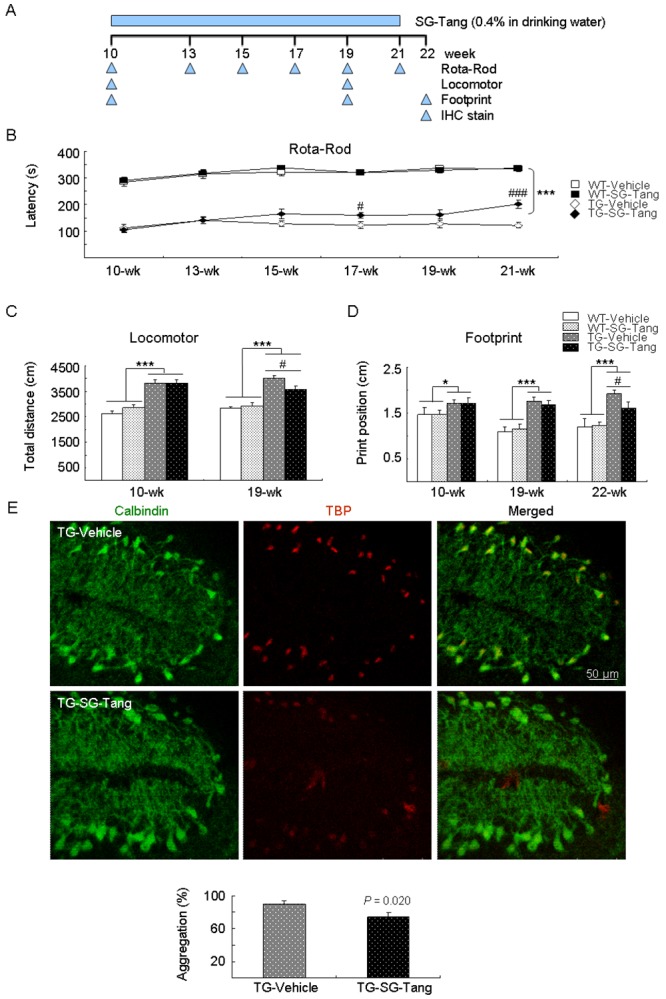
**Neuroprotective effect of SG-Tang in SCA17 mice.** (**A**) Experimental timeline. Mice received vehicle or SG-Tang (0.4% in drinking water) from 10 to 21 weeks old. Behavioral analyses were performed during (rotarod task) as well as at the beginning and the end (locomotor and footprint tasks) of the period to evaluate the treatment effect. In addition, IHC staining with 1TBP18 was performed at week 22. (**B**) Rotarod performance of wild type (WT) and SCA 17 transgenic (TG) mice at weeks 10, 13, 15, 17, 19, and 21 (*n* = 15; ^#^: *P* < 0.05, ^###^ or ***: *P* < 0.001). (**C**) Locomotor analysis of WT and SCA 17 TG mice at weeks 10 and 19 (*n* = 16; ^#^: *P* < 0.05, ***: *P* < 0.001). (**D**) Footprint analysis of WT and SCA 17 TG mice at weeks 10, 19, and 21 (*n* = 14; ^#^ or *: *P* < 0.05, ***: *P* < 0.001). (**E**) IHC staining of TBP aggregates (red) in calbindin-positive Purkinje cells (green) of SCA17 TG mice at week 22 (*n* = 3).

Lastly, we examined the neuroprotective effect of SG-Tang on an established SCA17 TBP/Q_109_ TG mouse model [33]. The experimental timeline is shown in Figure 5A. SG-Tang (0.4%) was supplied in the drinking water of SCA17 mice from 10 to 21 weeks old. To identify the effect of SG-Tang on motor function, rotarod, locomotor, and footprint tasks were performed. On the rotarod task to test motor coordination of mice, the SCA17 TG mice showed significantly decreased latency to fall at 10–21 weeks of age when compared with the WT mice (111 s–140 s versus 284 s–337 s, *P* < 0.001). However, the SG-Tang-treated TG mice showed significantly increased latency compared with the vehicle-treated TG mice at 17 weeks (159 s versus 123 s, *P* = 0.030) and 21 weeks (201 s versus 121 s, *P* < 0.001) of age (Figure 5B). On the locomotor task to test motor activity of mice, the SCA17 TG mice showed significantly increased total distance traveled in an open field (hyperactivity) at 10 weeks (3814 cm versus 2627 cm, *P* < 0.001) and 19 weeks (4003 cm versus 2830 cm, *P* < 0.001) of age when compared with the WT mice. However, the hyperactivity of TG mice was ameliorated when they were 19 weeks old after SG-Tang treatment (3576 cm versus 4003 cm, *P* = 0.015) (Figure 5C). On the footprint task to test gait coordination of mice, the SCA17 TG mice showed significantly increased print position at 10 weeks (1.7 cm versus 1.5 cm, *P* = 0.022), 19 weeks (1.8 cm versus 1.1 cm, *P* < 0.001), and 22 weeks (1.9 cm versus 1.2 cm, *P* < 0.001) of age when compared with the WT mice. However, SG-Tang treatment significantly improved print position in the TG mice compared with vehicle treatment at 22 weeks of age (1.6 cm versus 1.9 cm, *P* = 0.048) (Figure 5D). It is noticed that the rotarod performance of SG-Tang treated TG mice at 19 weeks of age showed a trend toward prolonged latency on rod compared with the vehicle-treated TG mice, but the improvement did not reach significance. This result may be due to the relatively small sample size with large variations in motor performance of tested TG mice. These results suggest that SG-Tang treatment ameliorated behavioral deficits in SCA17 TBP/Q_109_ TG mice.

In humans, the cerebellum plays an important role in motor control and is most adversely affected by SCAs. Calbindin in cerebellar Purkinje cells is a crucial determinant in motor functions [38]. We thus examined the cerebellar Purkinje cells in vehicle- or SG-Tang-treated SCA17 TG mice at 22 weeks of age using calbindin staining. As shown in Figure 5D, the disrupted alignment and structure of Purkinje cells in SCA17 TBP/Q_109_ TG mice was improved with the SG-Tang treatment, accompanied with reduced TBP aggregation (from 90% to 74%, *P* = 0.020).

## DISCUSSION

SCA17 is one of the neurodegenerative diseases caused by accumulation of expanded polyQ, the effective treatments for which are lacking. The elucidation of pathogenic mechanisms is important for development of therapeutics. Studies have shown PGC-1α/SOD2/CYCS and NRF2/GCLC/NQO1 pathways are involved in pathogenesis of polyQ-mediated diseases including HD and SCA3 [18,20,23,24], and impaired NFYA/HSPA5 expression has been shown in SCA17, including cellular and animal models and lymphoblasts from patients [6,7,9]. PGC-1α/SOD2/CYCS, NRF2/GCLC/NQO1, and NFYA/HSPA5 pathways may therefore serve as the therapeutic targets for SCAs. SG-Tang is composed of *P. lactiflora* and *G. uralensis*, both of which have been used as a traditional medicine to treat certain illnesses for their antioxidative, anti-inflammatory and cytoprotective properties. *P. lactiflora* or its constituent paeoniflorin have been shown to exert the protective effects in different rodent models of Alzheimer’s disease, the 6-OHDA-lesioned rodent model of Parkinson’s disease, and the SCA3 cell model [28,39–42]. *G. uralensis* and its bioactive compounds have displayed anti-inflammatory and antioxidative activities *in vitro* and *in vivo* [43–45]. SG-Tang has been used to inhibit inflammatory chemokine production in HaCaT cells [46]. Due to the multiple constituents in *P. lactiflora* and *G. uralensis* and their effects on different pathways, we set out to investigate if SG-Tang is beneficial to SCA17, the pathogenic pathways of which are also multiple. We also explored how SG-Tang acts on the underlying pathogenic pathways.

It is evident that the SG-Tang has a low cytotoxicity on uninduced and induced TBP/Q_79_-GFP 293 cells (as indicated by IC_50_ > 1 mg/ml in Figure 1) with the effective aggregation-inhibitory concentrations ranged from 0.001 to 100 μg/ml. Since the expression of TBP was not changed by SG-Tang, the aggregation-inhibitory effect may be related to decreased ROS rather than reduced TBP expression. Similarly, a very low cytotoxicity of SG-Tang on uninduced TBP/Q_79_-GFP SH-SY5Y cells at doses 0.1−100 μg/ml was shown (Figure 2A). The aggregation-inhibitory and neurite outgrowth promoting effects of SG-Tang at dose 100 μg/ml were further demonstrated in TBP/Q_79_-GFP-expressing SH-SY5Y cells (Figure 2D). Our *in vitro* result of low cytotoxicity of SG-Tang on TBP/Q_79_-GFP-expressing 293 and SH-SY5Y cells is consistent with previous studies suggesting its low cytotoxicity on ΔK280 tau_RD_-expressing 293 and SH-SY5Y cells [31] or HaCaT human keratinocytes [46], but some adverse effects of the main constituents have been shown. For example, paeoniflorin and albiflorin, two constituents of *P. lactiflora*, markedly suppressed the CYP3A4 and CYP2D6 activity [47]. In addition, paeoniflorin caused some allergic skin reactions [48]. *G. uralensis* was reported to significantly alter the metabolic rates of anticoagulants or antiplatelet drugs [49]. Glycyrrhizin, a main constituent of *G. uralensis*, at 1200–2600 mg/kg through oral intake displayed hypertension, polydipsia, bracycardia, and behavioral changes in rats [50]. These potential side effects should be further investigated by *in vivo* studies.

We further dissected the underlying protective mechanism of SG-Tang and showed that expression levels of PGC-1α/SOD2/CYCS, NRF2/GCLC/NQO1, and NFYA/HSPA5 were reduced in TBP/Q_79_-GFP-expressing SH-SY5Y cells, which were rescued by SG-Tang (Figure 3). Next, knockdown of PGC-1α, NRF2, or NFYA significantly attenuated the outgrowth-promoting effect of SG-Tang on TBP/Q_79_-GFP-expressing SH-SY5Y cells (Figure 4). This suggests that SG-Tang exerts the neuroprotective effect via enhancing PGC-1α/SOD2/CYCS, NRF2/GCLC/NQO1, and NFYA/HSPA5 pathways. Defects in mitochondrial biogenesis and function are common in polyQ-mediated diseases including HD and SCAs and enhancement of mitochondrial biogenesis or function can protect against polyQ-induced neurodegeneration [18,23,24]. Since PGC-1α is an important regulator of mitochondria biogenesis and antioxidative response, enhancement of PGC-1α expression has been implicated to be a potential therapeutic strategy for polyQ-mediated and other neurodegenerative diseases [51–53]. Our study has provided evidence that augmented expression of PGC-1α, SOD2, and CYCS protects against neurotoxicity of expanded polyQ in the SCA17 cell and mouse models.

NRF2, a transcription factor that regulates expression of many cytoprotective genes, mediates redox adaptations to exercise as well as defendant responses to oxidative stress [19,54]. Since oxidative stress may lead to neurodegeneration, NRF2 activators have been suggested to be a therapeutic target for the treatment of neurodegenerative diseases [55]. NRF2 enhancers have been applied to treat different models of polyQ diseases [18,56–58]. Our study results further support the potential of NRF2 activators for treating SCA17 and other polyQ diseases.

The trimeric NFY is formed by three subunits, NFYA, NFYB, and NFYC. The sequence specific interactions of the complex are made by the NFYA subunit, suggesting its role as the regulatory subunit [59]. Recent studies have shown that mutant huntingtin aggregates sequester NFYA and NFYC leading to the reduction of HSPA1A gene expression in the brain of a HD mouse model, indicating NFY components as modulators of the HD pathological process [60]. Similarly, mutant TBP with expanded polyQ interacts aberrantly with NFYA, which impairs the transcriptional function [6,7,61]. Because NFYA is a key regulator of HSPA5 transcription [62] and downregulated HSPA5 has been shown in SCA17 lymphoblasts [9], treatments through enhancing NFYA expression may be beneficial to SCA17. Our results demonstrate that SG-Tang rescues downregulated NFYA and HSPA5 in SCA17 cell model.

According to the antiaggregation effect on SCA17 SH-SY5Y cells (Fig. 2), the effective dosage for SG-Tang was 100 μg/ml. To assume the bioactivity of SG-Tang as 10% and protein binding rate as 90%, the dosage for treating animals can be roughly estimated to be 10 mg/25 g/day or 0.4 g/kg/day per mouse. The SCA17 transgenic mice performed poorly on an accelerating rotarod around 2 and 4 months of age and showed continued declines in latency on the rod [33]. Thus 10 weeks of age was selected to receive 0.4% SG-Tang in drinking water for 12 weeks. Average daily consumption of water for these mice was 3 ml. The dosage of 3 ml 0.4% SG-Tang administered to SCA17 mouse in the present study would be equivalent to 0.48 g/kg/day per mouse. The dosage of SG-Tang for a 50 kg human adult to alleviate pain recommended by the Sun Ten Pharmaceutical Co. Ltd. is 4 g 3 times daily, or roughly 0.24 g/kg/day. The dosage of SG-Tang used to treat SCA17 mice was about twice amount used to relieve pain in human. Further experiments will need to be carefully designed to examine the possible adverse effects of SG-Tang in SCA17 mice and optimal dosage for future clinical application.

There are several limitations in our study. Since there are multiple constituents in SG-Tang, the main components or compounds responsible for the protective effects in our cellular models are not known. However, paeoniflorin, the bioactive compound of *P. lactiflora* demonstrating protective effects through activating the NRF2/ARE pathway in other cell models of oxidative injury has been shown [63,64]. The bioactive compounds of *G. uralensis* such as licochalcone A, glycyrrhetinic acid, liquiritigenin, isoliquiritigenin and liquiritin were found to be all potent NRF2 or NQO1 inducers [18,65,66]. Paeoniflorin also protected Aβ_25-35_-expressed SH-SY5Y cells from cytotoxicity by preventing mitochondrial dysfunction [67]. Previously we also showed that licochalcone A and ammonium glycyrrhizinate could activate PGC-1α activity in a SCA3 cell model [18]. We therefore propose that the effects of PGC-1α, NRF2, and NFYA enhancement may be mediated by paeoniflorin, licochalcone A, ammonium glycyrrhizinate, glycyrrhetinic acid, liquiritigenin, isoliquiritigenin, or liquiritin in SG-Tang. Future studies examining how individual bioactive compounds of SG-Tang exert the specific effects on the PGC-1α/SOD2/CYCS, NRF2/ GCLC/NQO1, and NFYA/HSPA5 pathways are necessary to consolidate our results and clarify the therapeutic mechanism. Besides, there are protective effects of SG-Tang through other pathways not examined in the present study, which requires further investigation in the future. Since the main pathology of SCA17 is in brain, the most important prerequisite for a good therapeutic agent for treating neurodegenerative diseases is its high blood brain barrier (BBB) permeability. Previous studies have shown a good BBB permeability of isoliquiritigenin and 18β-glycyrrhetinic acid, a major metabolite of glycyrrhizin in *G. uralensis* [68–70]. Our studies also have another limitation that the *in vitro* models did not show the BBB permeability of SG-Tang. It will facilitate proceeding of clinical trials of SG-Tang in SCA17 and other polyQ diseases if efficient BBB permeability of SG-Tang and its main bioactive compounds can be shown in the future animal studies. Finally, although we have shown the improved motor performance and decreased cerebellar aggregates in SCA17 mice treated with SG-Tang, we did not investigate how SG-Tang exerts its neuroprotection effect on mice. In the future, examining the PGC-1α/SOD2/CYCS, NRF2/GCLC/NQO1, and NFYA/ HSPA5 pathways in SCA17 mice treated with specific constituents of SG-Tang is warranted to uncover the underlying mechanisms.

**Figure 6:**
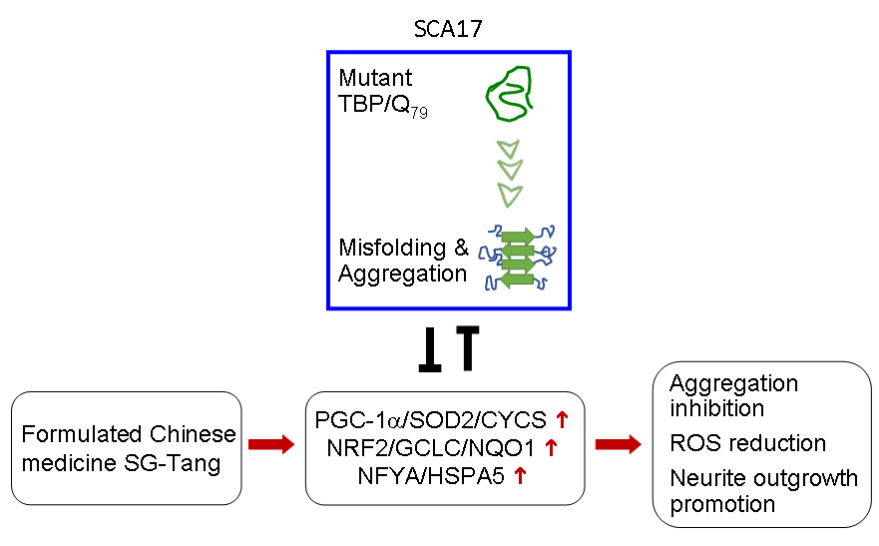
Graphic summary.

In conclusion, we have provided evidence that PGC-1α/SOD2/CYCS, NRF2/GCLC/NQO1, and NFYA/ HSPA5 pathways are downregulated in SCA17 cell models. We further demonstrated that SG-Tang may serve as a protective agent for SCA17 via upregulating the PGC-1α/SOD2/CYCS, NRF2/GCLC/NQO1, and NFYA/HSPA5 pathways (Figure 6). Based on these results, future work is warranted to uncover the main constituents in this Chinese medicine formula and its adverse effects on human health.

## MATERIALS AND METHODS

### Formulated SG-Tang and its ingredients

SG-Tang (Code: 0703H), provided by Sun Ten Pharmaceutical Co. Ltd. (New Taipei City, Taiwan), is a formula in the traditional Chinese medical Shang Han Lun. SG-Tang consisting of two Chinese herbs Paeoniae Radix Alba and Glycyrrhizae Radix et Rhizoma (use Honey baked) at 1:1 (w/w) ratio. The botanical origins of each ingredient are *Paeonia lactiflora* Pall. and *Glycyrrhiza uralensis* Fisch., harvested in An Hui (*Paeonia lactiflora* Pall.) and Inner Mongolia (*Glycyrrhiza uralensis* Fisch.), China, respectively. These materials had passed the strict quality tests with methods and acceptance criteria based upon the requirements of Chinese pharmacopoeia. Quality control factors include macroscopic identification, microscopic identification, thin layer chromatographic identification, loss on drying, total ash, acid-insoluble ash, water soluble extract, dilute ethanol soluble extract, high performance liquid chromatography fingerprint, marker substance assay, residual pesticides, heavy metals, sulfur dioxide, aflatoxins, and microbiological contaminants. The SG-Tang is manufactured by decocting (boiled with one hour), concentrating, fluid-bed granulating, and sieving. Decocted intermediate is granulated with corn starch and cellulose as diluents and magnesium stearate as a glidant. The certificate of analysis for finished product includes physical description, thin layer chromatographic identification, loss on drying, total ash, acid-insoluble ash, water soluble extract, dilute ethanol soluble extract, particle size examination, weight conformity, heavy metals (arsenic, cadmium, mercury, and lead) contaminants, and microbiological (total viable aerobic count, *Escherichia coli*, and *Salmonella*) contaminants with suitable specifications accordingly. The certificate of analysis is available on request.

### Cell culture and cell proliferation assay

Two human cell lines, Tet-On TBP/Q_79_-GFP 293 and SH-SY5Y cells [32] were used. Cells were cultivated in Dulbecco’s modified Eagle’s medium (DMEM) containing 10% fetal bovine serum (FBS) (Invitrogen, Carlsbad, CA, USA) with addition of blasticidin (5 µg/ml) and hygromycin (100 µg/ml) (InvivoGen, San Diego, CA, USA) at 37°C under 5% CO_2_ and 95% humidity. To evaluate cell viability, 5 × 10^4^ cells were plated on 48-well dishes, grown for 20 h, and treated with the formulated SG-Tang (0.1−100 μg/ml). After 1 day, 20 μl of MTT (5 mg/ml) was added to the cells at 37°C for 2 h. 200 μl of lysis buffer (10% Triton X-100, 0.1 N HCl, 18% isopropanol) was then added to dishes and the absorbance of the insoluble purple formazan product at OD 570 nm was read by a FLx800 fluorescence microplate reader (Bio-Tek, Winooski, VT, USA).

### 293 TBP/Q_79_ aggregation assay

GFP fluorescence was evaluated to reflect TBP aggregation in TBP/Q_79_-GFP-expressing 293 cells. Briefly, cells were plated on 96-well (2 × 10^4^/well) dishes, grown for 24 h and treated with different concentrations of SG-Tang (0.001−1000 μg/ml), or a positive control suberoylanilide hydroxamic acid (SAHA, 0.1 µM) (Cayman Chemical, Ann Arbor, MI, USA). After 8 h, doxycycline (10 µg/ml) and oxaliplatin (5 µM) (Sigma-Aldrich, St Louis, MO, USA) were added to the cells for 6 days to induce TBP/Q_79_-GFP expression and inhibit cell cycle progression [71], respectively. The cells were kept in the medium containing doxycycline, oxaliplatin and SG-Tang for 6 days. On the eighth day, cells were stained with Hoechst 33342 (0.1 µg/ml; Sigma-Aldrich) for 30 min, and images of the cells were automatically obtained using an ImageXpressMICRO high content analysis (HCA) system (Molecular Devices, San Jose, CA, USA) (482 nm excitation and 536 nm emission for enhanced GFP; 377 nm excitation and 447 nm emission for Hoechst 33342). Aggregation was determined by Transfluor technology [72] based on GFP fluorescence intensity. To quantify aggregation, the relative aggregation level in untreated cells was set as 100%. In addition, IC_50_ value (half-maximal inhibitory concentration) for SG-Tang was evaluated based on the survived cell number.

### ROS analysis

293 TBP/Q_79_-GFP cells were plated on 6-well (5 × 10^4^/well) dishes, treated with the SG-Tang (100 μg/ml) for 8 h, and induced TBP/Q_79_-GFP expression for 6 days. Fluorogenic CellROX deep red reagent with final concentration of 5 μM (Molecular Probes, Eugene, OR, USA) was added to the cells and incubated at 37°C for 30 min. Then the cells were washed with PBS and analyzed by a flow cytometer (Becton-Dickinson, Franklin Lakes, NJ, USA) with excitation/emission wavelengths at 488/507 nm (green, TBP/Q_79_-GFP expression) and 640/665 nm (red, ROS). For each sample, 5 × 10^4^ cells were analyzed.

### SH-SY5Y TBP/Q_79_-GFP aggregation and neurite outgrowth assays

2 × 10^4^ of TBP/Q_79_-GFP SH-SY5Y cells were seeded on a 24-well plate, and retinoic acid (10 µM; Sigma-Aldrich) was added to initiate neuronal differentiation. On the second day, cells were treated with SG-Tang (100 µg/ml) for 8 h before TBP/Q_79_-GFP expression induction by adding doxycycline (5 µg/ml). The cells were kept in the medium containing retinoic acid, doxycycline and SG-Tang for 6 days. On day 8, cells were stained with Hoechst 33342 (0.1 µg/ml) and the aggregation percentage was assessed by HCA as described. In addition, the morphologic differentiation of TBP/Q_79_-GFP-expressing cells was assessed by using Metamorph microscopy automation and image analysis software (neurite outgrowth application module, Molecular Devices). To quantify neurite outgrowth, the relative outgrowth in untreated cells was set as 100%.

### Western blot analysis

Cells were lysed using buffer (50 mM Tris-HCl pH8.0, 150 mM NaCl, 1 mM EDTA pH8.0, 1 mM EGTA pH8.0, 0.1% SDS, 0.5% sodium deoxycholate, 1% Triton X-100) containing the protease inhibitor mixture (Sigma-Aldrich). After sonication, the lysates were centrifuged at 15,400 × g for 5 min at 4°C. Protein concentrations were determined using a protein assay kit (Bio-Rad, Hercules, CA, USA), with albumin as standards. Total proteins (20 µg) were electrophoresed on 10%−12% SDS-polyacrylamide gel and transferred onto nitrocellulose membrane (Schleicher and Schuell, Dassel, Germany) by reverse electrophoresis. After being blocked, the membrane was stained with PGC-1α, SOD2, NRF2, HSPA5, NFYA (1:500; Santa Cruz Biotechnology, Santa Cruz, CA, USA), CYCS (1:1000; BioVision, Milpitas, CA, USA), GCLC (1:1000; Abcam, Cambridge, MA, USA), NQO1 (1:1000; Sigma-Aldrich), or GAPDH (1:1000; MDBio Inc., Taipei, Taiwan) primary antibody at 4°C overnight. The immune complexes were detected using horseradish peroxidase-conjugated goat antimouse or goat antirabbit IgG antibody (1:10000; GeneTex, Irvive, CA, USA) and chemiluminescent substrate (Millipore, Billerica, MA, USA).

### RNA interference

To knock down the expression of specific genes in TBP/Q_79_-GFP SH-SY5Y cells, small interfering RNA (siRNA) targeting PGC-1α (sc-38884), NRF2 (sc-37030), NFYA (sc-29947) and a scrambled control (sc-37007) (Santa Cruz Biotechnology) were used. Cells were plated at a density of 8 × 10^5^/well on 6-well plates in the presence of retinoic acid on day 1 as described. On day 2, the cells were transfected with siRNA (100 nM) using T-Pro NTR II reagent (3 µl) (T-Pro Biotechnology, New Taipei City, Taiwan). Twenty-four hours post-transfection, the culture medium was changed and the cells were pretreated with SG-Tang (100 µg/ml) for 8 h followed by inducing TBP/Q_79_-GFP expression with doxycycline (5 µg/ml) for 6 days. Cells were then collected for PGC-1α, NRF2, and NFYA protein analysis or stained with Hoechst 33342 and analyzed for aggregation and neurite outgrowth as described.

### SCA17 mice and SG-Tang treatment

Previously, SCA17 transgenic (TG) mice were established with Purkinje cell-specific Pcp2 promoter driving the expression of human TBP/Q_109_ [33]. SCA17 TG mice and their wild-type littermates (WT) were kept in individually ventilated cages (Lasco, Taipei, Taiwan) under controlled temperature (25 ± 2°C), humidity (50%), and normal light/dark (12h/12h) cycle. Mice were maintained *ad libitum* on food and water at the Animal House Facility of National Taiwan Normal University (NTNU). At age of 3 weeks old, both WT and TG mice were randomly divided into vehicle and SG-Tang groups (*n* = 18 in each group). From 10–21 weeks of age, mice in SG-Tang group received 0.4% SG-Tang in drinking water for 12 weeks. Behavioral analyses were performed during (rotarod task) and at the beginning and near the end (locomotor and footprint tasks) of the period to evaluate the treatment effect. All procedures were conducted in compliance with the ARRIVE (Animal Research: Reporting *In Vivo* Experiments) guidelines and approved by the Institutional Animal Care and Use Committee of NTNU (Permit Number: 103002).

### Rotarod, locomotor, and footprint tasks

The motor coordination of WT and TG mice was analyzed by rotarod (Ugo Basile, Comerio, VA, Italy) from 10 to 21 weeks of age. Mice were tested starting at 4 rpm and accelerating to 30 rpm over a period of 5 min and then maintaining a constant speed of 30 rpm for 1 min. The time (latency) it takes the mouse to fall off the rod was recorded. The latencies of four trials given on 2 days were averaged as the performance of each mouse.

For locomotor activity monitoring, 10- and 19-week-old mice were placed in the center of an open field apparatus (30 × 30 × 30 cm) and monitored under low lighting (2 lux) for 10 min. An EthoVision system (Noldus, Wageningen, Netherlands) was used to record and analyze the distance traveled.

Mouse footprints were monitored when the mice were 10, 19, and 22 weeks old using the CatWalk XT system (Noldus). Each mouse was allowed to walk three times on the glass plate, with paw prints recorded and analyzed using the CatWalk XT 9.1 software (Noldus).

### Immunohistochemistry stain

At the age of 22 weeks, TG mice were perfused with 0.9% NaCl followed by 4% paraformaldehyde (Sigma-Aldrich) after fully anesthetized with avertin (0.4 g/kg body weight; Sigma-Aldrich). The whole mouse brain was then postfixed in 4% paraformaldehyde for 4 h at 4°C, followed by a series of dehydration in sucrose gradient for 1 h (10% sucrose) to overnight (20% and 30% sucrose). Then cerebellum tissue was cut in 30 μm thick slices by a CM3050S cryostat microtome (Leica Biosystems, Nussloch, Germany). After incubating with primary antibody calbindin (1:1000; Santa Cruz Biotechnology) or 1TBP18 (1:3000; QED Bioscience, San Diego, CA, USA) at 4°C overnight and washing three times for 10 min in TBST (PBS containing 2% Triton X-100), the cerebellar slices were incubated with secondary antibodies (1:500; Alexa Fluor dye-conjugated donkey antimouse or antigoat IgG, Invitrogen) for 2 h at room temperature and nuclei stained with 4’-6-diamidino-2-phenylindole (DAPI) (1:10000; Sigma-Aldrich). Finally, the stained cerebellar slices were mounted on gelatin-coated slides for observation under a LSM 880 confocal microscope (Zeiss, Oberkochen, Germany). In each experiment, 4–6 slices per mouse were analyzed with *n* = 3 per group. The nuclear TBP aggregation in Purkinje cells was represented by the colocalization with calbindin staining.

### Statistical analysis

For each data set, the experiments are performed three times and data were expressed as the means ± standard deviation (SD). Differences between groups were evaluated by Student’s *t* test (comparing two groups) or one way analysis of variance with a *post hoc* LSD test where appropriate (comparing several groups). All *P* values were two-tailed, with values lower than 0.05 to be considered statistically significant.

## SUPPLEMENTARY MATERIAL

Supplementary Figures
