# Supplementary material for: Shaoyao Gancao Tang (SG-Tang), a formulated Chinese medicine, reduces aggregation and exerts neuroprotection in spinocerebellar ataxia type 17 (SCA17) cell and mouse models
Source: Aging (Albany NY). 2019 Feb 13;11(3):986–1007. doi: 10.18632/aging.101804 (PMC6382417; doi:10.18632/aging.101804)
Supplement: Supplementary Figures [file aging-11-101804-s001.pdf]

SUPPLEMENTARY MATERIAL

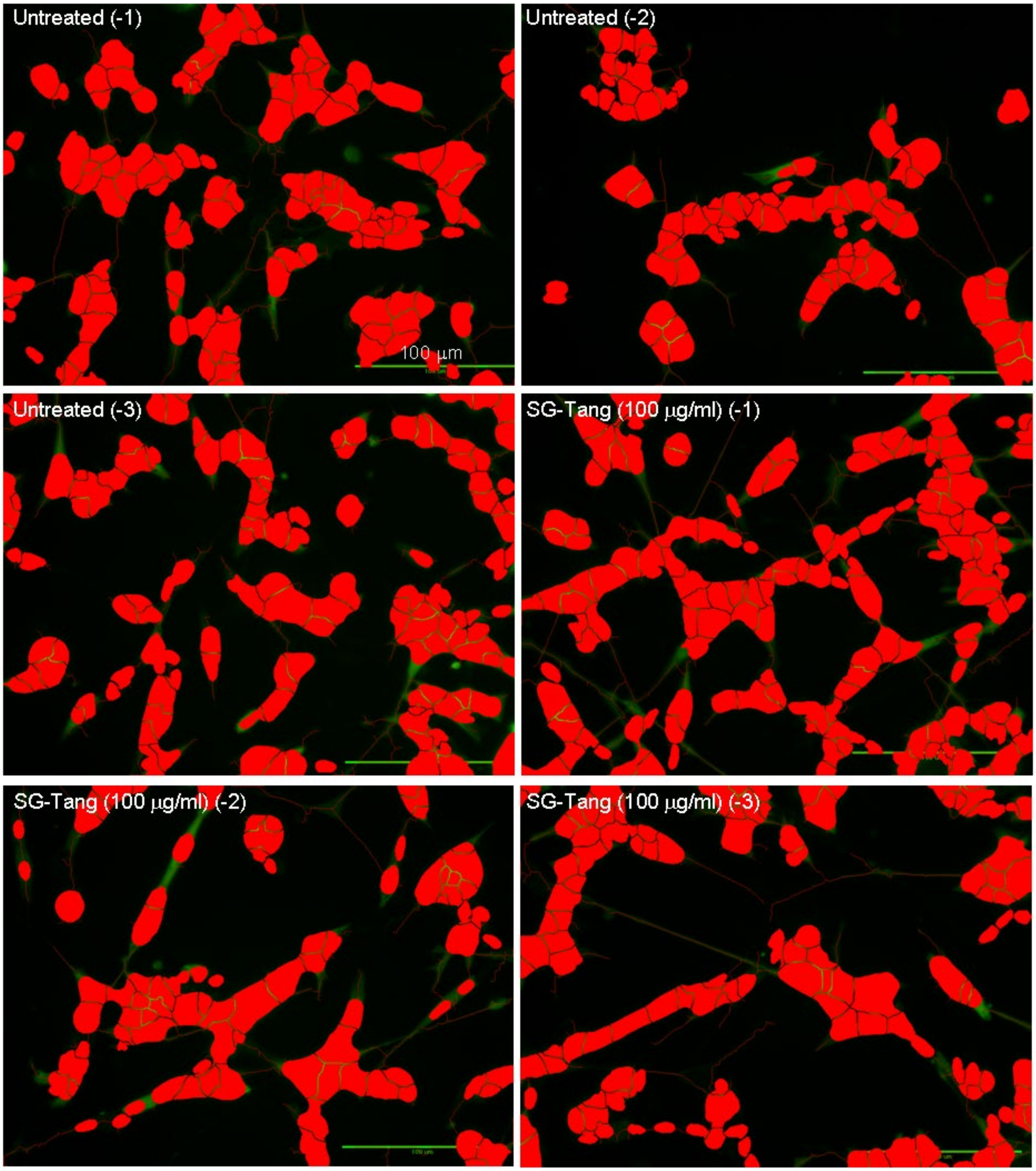

Supplementary Figure 1. Microscopic images of differentiated TBP/Q_{79}-GFP SH-SY5Y cells untreated or treated with SG-Tang (100 µg/ml) in triplicate (-1, -2, and -3), with neurites and cell bodies being outlined by red color for outgrowth quantification.

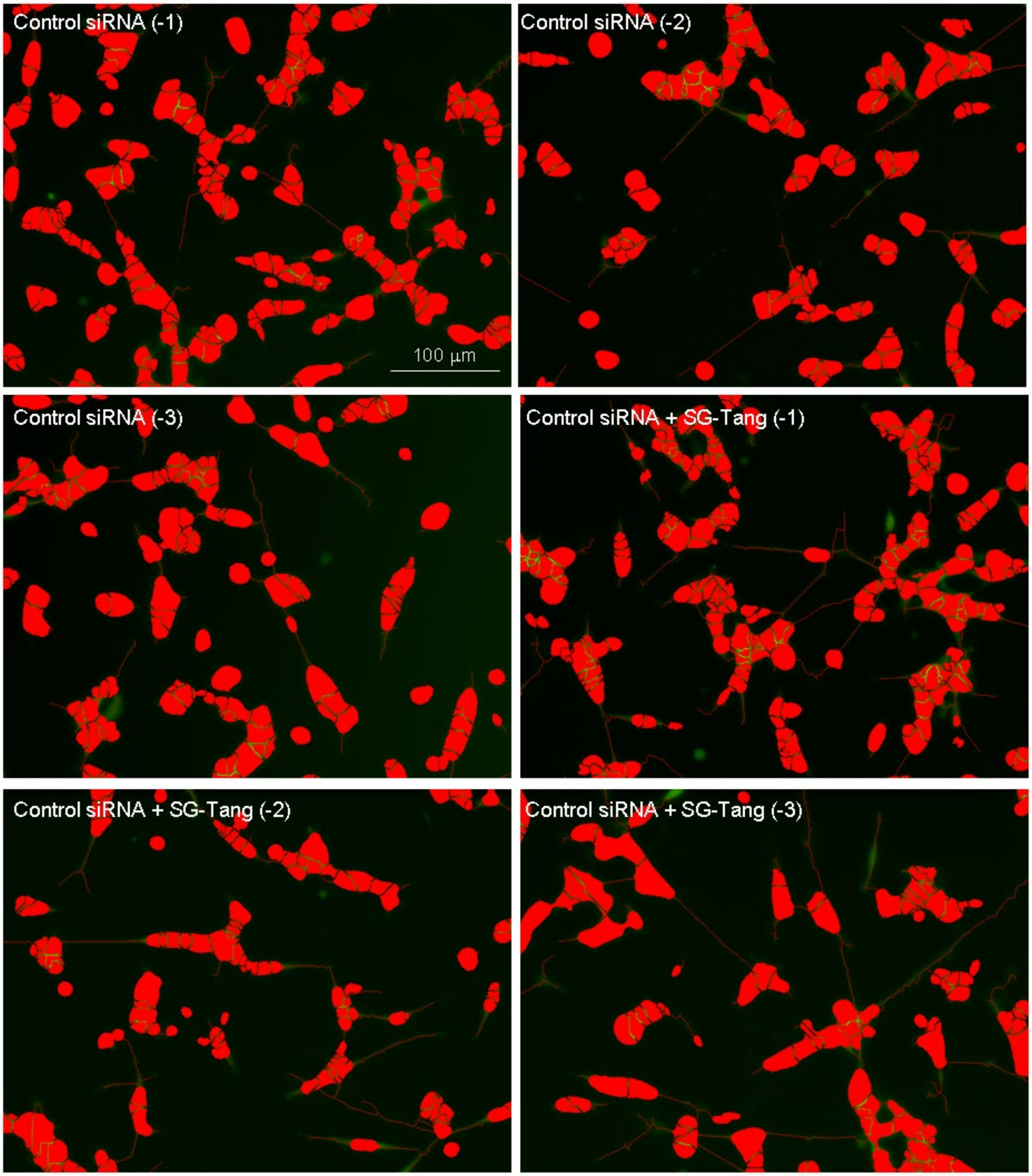
Control siRNA (-1)
100 µm
Control siRNA (-2)
Control siRNA (-3)
Control siRNA + SG-Tang (-1)
Control siRNA + SG-Tang (-2)
Control siRNA + SG-Tang (-3)

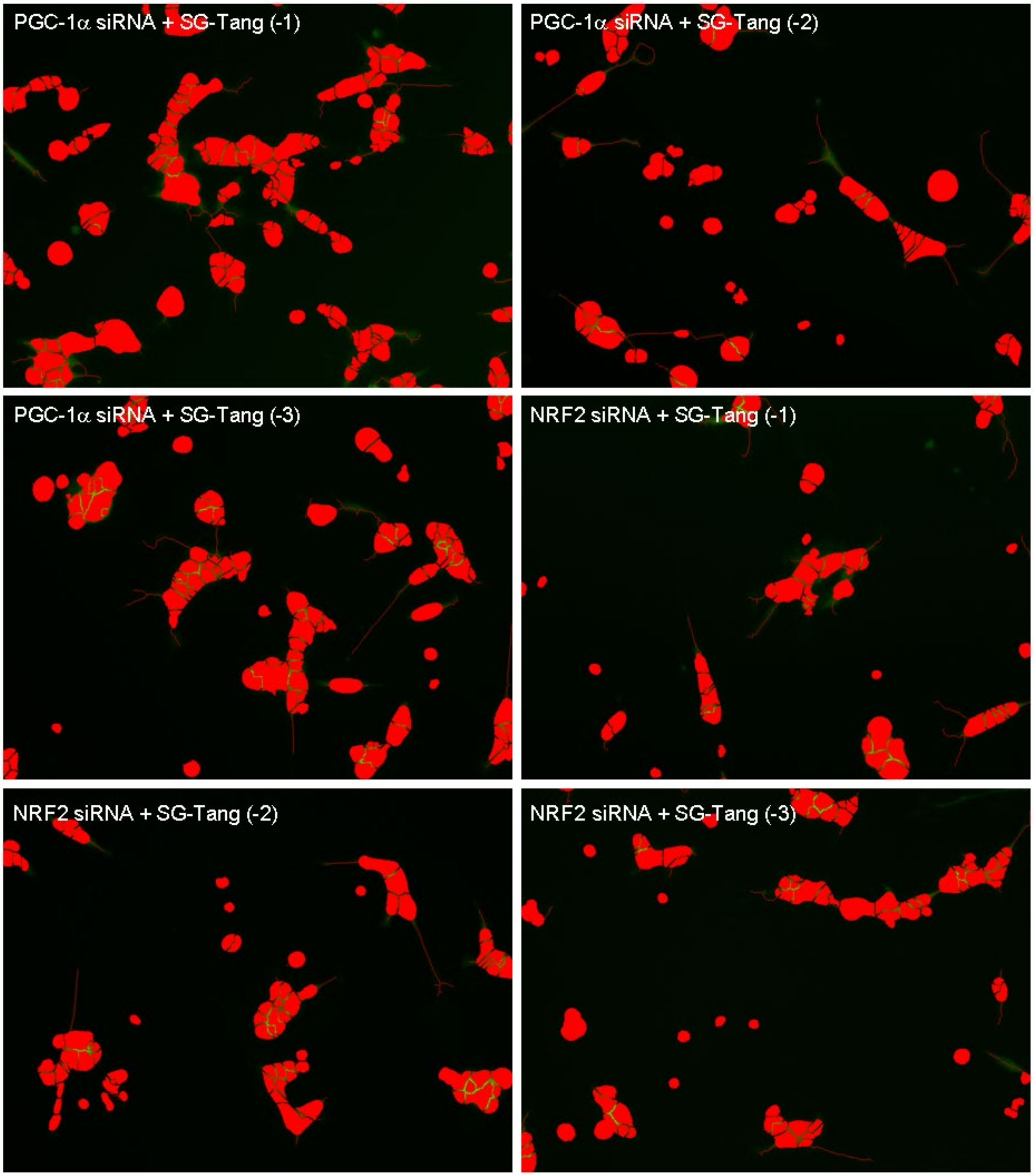
PGC-1α siRNA + SG-Tang (-1)
PGC-1α siRNA + SG-Tang (-2)
PGC-1α siRNA + SG-Tang (-3)
NRF2 siRNA + SG-Tang (-1)
NRF2 siRNA + SG-Tang (-2)
NRF2 siRNA + SG-Tang (-3)

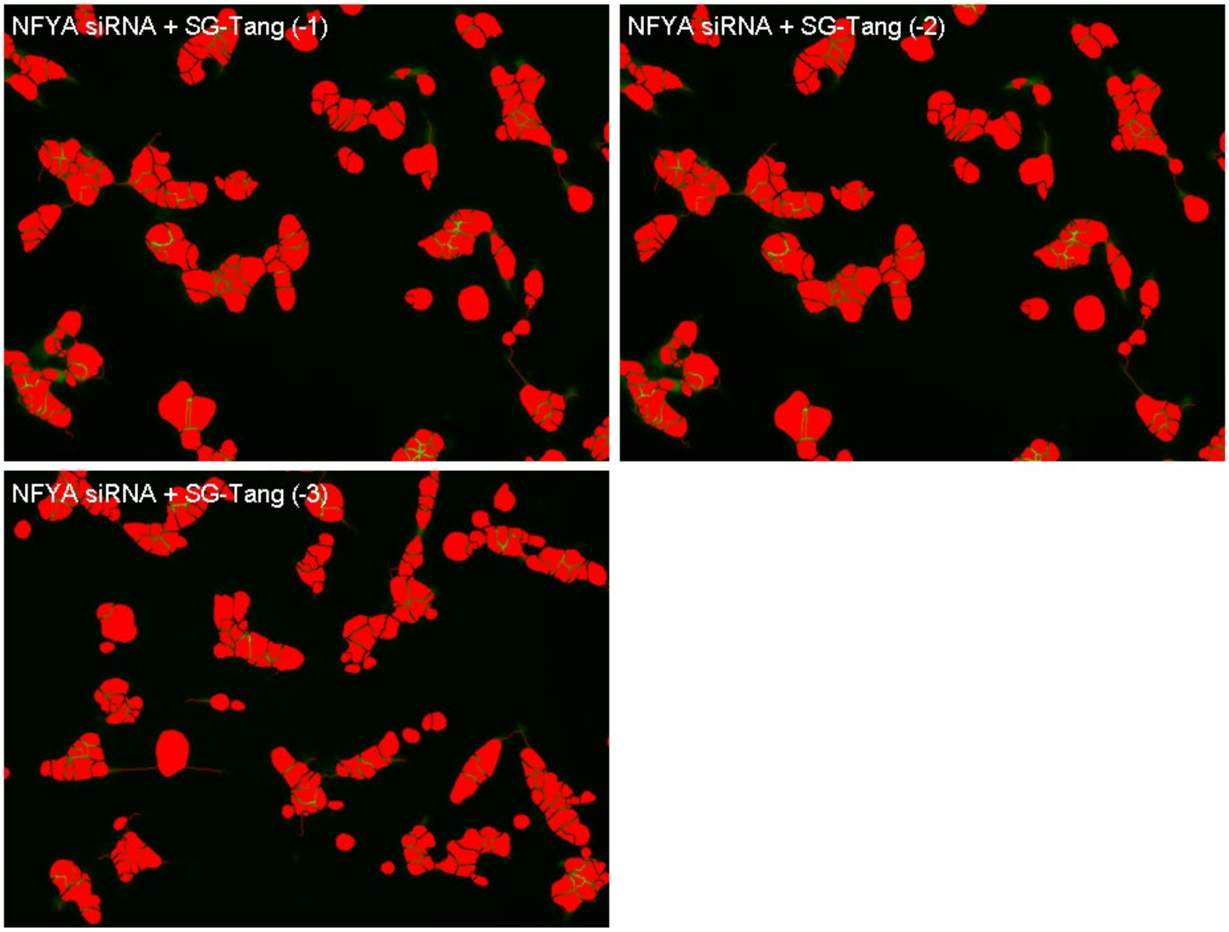

Supplementary Figure 2. Microscopic images of TBP/Q_{79}-GFP SH-SY5Y cells transfected with scrambled control siRNA and SG-Tang-treated cells transfected with scrambled control, PGC-1α, NRF2, or NFYA siRNA in triplicate (-1, -2, and -3). Neurites and cell bodies were outlined by red color for outgrowth quantification.
